# Why compliance and driving pressure may be inappropriate targets for PEEP setting during ARDS

**DOI:** 10.1186/s13054-022-04109-7

**Published:** 2022-08-02

**Authors:** Domenico Luca Grieco, Filippo Bongiovanni, Antonio M. Dell’Anna, Massimo Antonelli

**Affiliations:** 1 Department of Emergency, Intensive Care Medicine and Anesthesia, Fondazione Policlinico Universitario A. Gemelli IRCCS, L.go F. Vito, 00168 Rome, Italy; 2 Istituto di Anestesiologia e Rianimazione, Università Cattolica del Sacro Cuore, Rome, Italy

We read with great interest the viewpoint by Cove and coworkers entitled “Are we ready to think differently about setting PEEP?”, recently published in the journal [1]. The authors provide an insightful explanation of the unresolved issue of positive end-expiratory (PEEP) setting in acute respiratory distress syndrome (ARDS) patients, addressing the pitfalls of the protocols foreseeing stepwise increases in PEEP proportional to the fraction of inspirated oxygen (FiO_2_) required to maintain arterial oxygen levels within a physiological range (the so-called PEEP-FiO_2_ tables) [2]. These PEEP-FiO_2_ tables are based on the concept that there is a substantial correlation between ARDS severity and lung recruitability [3]; due to their simplicity to use, they are often applied at the bedside and used as control strategy in randomized studies, especially as no other approach has been proven superior in terms of clinical outcome in randomized trials.

Indeed, lung recruitability as a response to PEEP has wide inter-subject variability. High PEEP in patients with poor recruitability increases static stress and strain and may induce right ventricular dysfunction, finally contributing to ventilator-induced lung injury and multi-organ dysfunction. Low PEEP in recruitable patients does not fully exert its potential benefits, which include avoidance of atelectrauma and reduction in the mechanical distortion provided by tidal volume in the aerated lung (i.e., the dynamic strain) [4]. Cove and coworkers propose that a PEEP-setting approach to target the highest respiratory system compliance (and lowest driving pressure), which can be easily measured on every mechanical ventilator, can identify the PEEP level that best benefits the patient. From authors’ perspective, if PEEP generates recruitment of functional lung units, compliance increases; conversely, if few or no functional units are recruited, compliance remains unchanged or decreases due to overdistension of already open lung tissue. This hypothesis comes from the classical physiological concept of proportionality between respiratory system compliance and aerated lung size [5].

The idea of personalizing PEEP based on the amount of individual recruitment is physiologically sound [6]. Unfortunately, raising evidence indicates that PEEP-induced changes in compliance and driving pressure are inaccurate measures of the amount of recruitment. This happens both in COVID-19 and non-COVID-19 ARDS.

Alveolar recruitment can be measured with different tools: Computed tomography scan provides information about the so-termed tissue recruitment; electrical impedance tomography, pressure–volume curves and their derived indices (as the recruitment-to-inflation ratio) measure gas recruitment in the lungs [7, 8].

In a recent computed tomography scan study, significant tissue recruitment was not systematically accompanied by increases in compliance, nor absence of recruitment could be identified by unchanged or reduced compliance [9]. Regarding gas recruitment, we re-analyzed data from a previously published study on 30 COVID-19 suffering from moderate-to-severe ARDS early after intubation [10]: Respiratory mechanics were measured at PEEP 15 and 5 cmH_2_O, and alveolar recruitment was measured through a simplified derecruitment maneuver. With constant tidal volume, change in PEEP from 5 to 15 cmH_2_O was associated with increased (> 5 ml/cmH_2_O) compliance in 6 patients (20%), unchanged compliance in 11 patients (37%) and reduced (< 5 ml/cmH_2_O) compliance in 13 patients (43%). Average alveolar recruitment was 32 ml per cmH_2_O of applied PEEP, and mean recruitment-to-inflation ratio was 0.81. As shown in Fig. 1, neither alveolar recruitment nor the recruitment-to-inflation ratio could be predicted by changes in respiratory system compliance (*p* = 0.58 and *p* = 0.14, respectively).

**Fig. 1:**
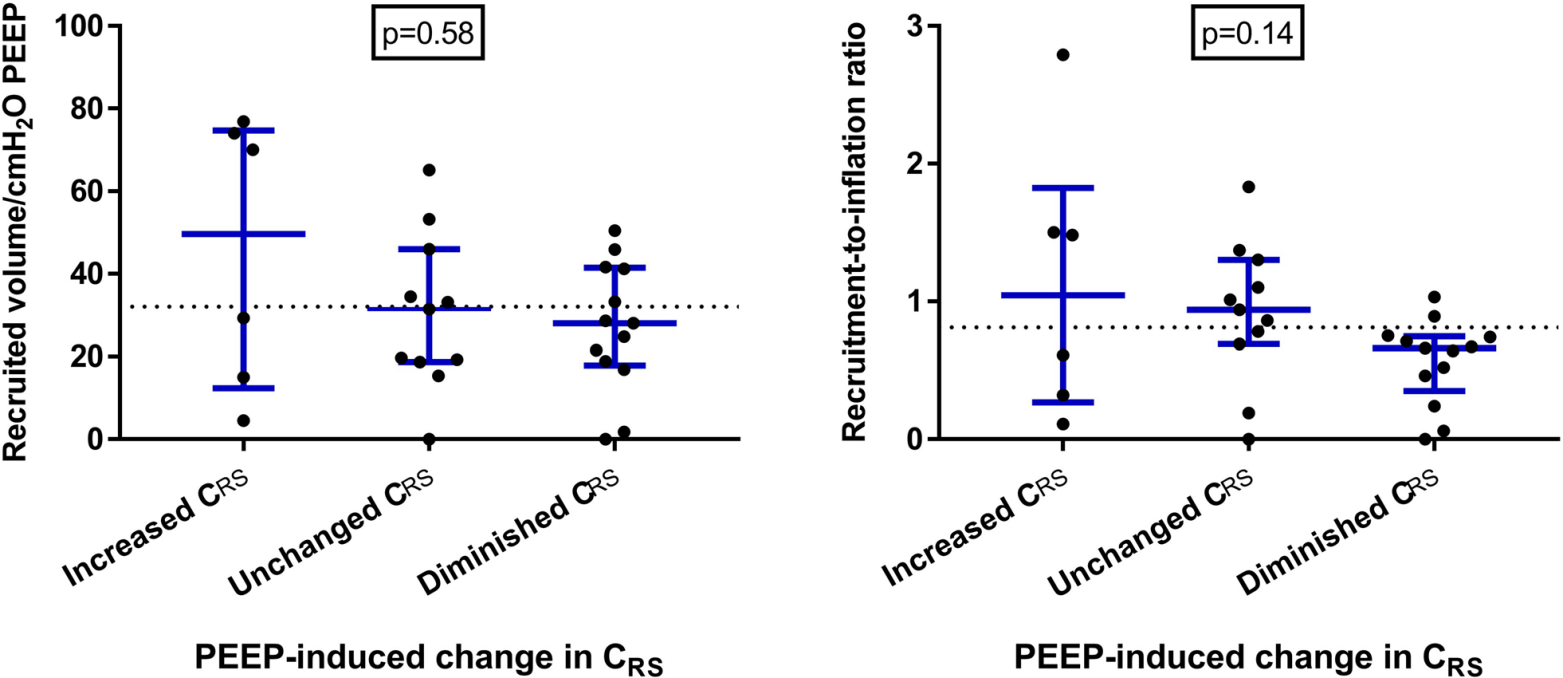
Potential for lung recruitment and changes in respiratory system compliance induced by PEEP in 30 ARDS patients. Re-analysis of data from [10]. Lung recruitability (assessed as absolute recruited volume normalized to the change in PEEP—left, and the recruitment-to-inflation ratio—right) in patients who showed increased, unchanged (defined as a clinically relevant modification of < 5 ml/cmH_2_O) or diminished respiratory system compliance as a response to 10-cmH_2_O PEEP increase. Changes in respiratory system compliance are ineffective measure of the recruitment induced by PEEP. Data from individual patients, medians and interquartile ranges are displayed. The dotted horizontal lines represent the mean values of recruitment and recruitment-to-inflation ratio in the study cohort. Data were analyzed with the Kruskal Wallis test. C_RS_ = respiratory system compliance

These data indicate that compliance and driving pressure are ineffective estimates of PEEP-induced lung recruitment, both if recruitment is assessed as tissue recruitment with computed tomography scan or as gas recruitment with pressure–volume curves-derived indices. Using compliance and driving pressure to assess the response to PEEP may seriously mislead clinicians.

There are several mechanisms that explain why the “physiologically sound” model foreseeing that changes in compliance reflect presence or absence of recruitment fails to work in clinical practice. First, compliance and driving pressure are global measures and do not account for the regional behavior of lung tissue; alveolar recruitment and overdistension are regional and heterogeneous phenomena. Second, tidal recruitment is a common phenomenon in ARDS patients, especially at low PEEP. Tidal recruitment is the cyclic opening and closing of alveolar units during tidal ventilation. When this occurs, alveolar units that are collapsed at end-expiration reopen due to the increase in airway pressure produced by tidal volume inflation. Respiratory system compliance is the sum of the compliance of each alveolar unit that is open at end-inspiration. When a collapsed alveolar unit reopens during inspiration, as it is in case of tidal recruitment, its individual compliance tends toward infinity. This finally increases static respiratory system compliance. This is why tidal recruitment makes static compliance very high at low PEEP and explains why increases in PEEP, which limits tidal recruitment, may generate worsening compliance also in case of significant recruitment. In our study [10], 80% of patients showed unchanged or decreased compliance in spite of alveolar recruitment within average values. Reduced compliance and increased driving pressure despite significant recruitment have been reported also in patients with ARDS of non-COVID-19 etiology [11].

Importantly, one large randomized trial on more than 1,000 patients showed that PEEP set to maximize respiratory system compliance (and limit driving pressure) may worsen patients’ survival, as compared to the low-PEEP-FiO_2_ table [12].

We fully agree with Cove and coworkers that the search for a personalized PEEP-setting strategy for moderate-to-severe ARDS patients is of utmost importance and represents a research priority. Assessment of respiratory system compliance and limiting driving pressure are essential to guide tidal volume setting [13]. Differently, clinical and physiological data do not support the use of a PEEP-setting strategy to target maximal respiratory system compliance (or minimal driving pressure) during ARDS.

From a clinical standpoint, setting PEEP with the aim of achieving a transpulmonary pressure close to 0 cmH_2_O seems promising in obese patients [14, 15]. Other physiology-based protocols providing individualized PEEP driven by lung recruitability assessment through electrical impedance tomography, recruitment-to-inflation ratio (NCT03963622) or bedside lung volume measurement (NCT04012073) are being tested in randomized trials and will hopefully illuminate the important aspect of PEEP individualization during ARDS.

## Data Availability

The datasets used and/or analyzed during the current study are available from the corresponding author on reasonable request.

## Abbreviations

ARDS: Acute respiratory distress syndrome
PEEP: Positive end-expiratory pressure
